# Prognostic effects of treatment protocols for febrile convulsive status epilepticus in children

**DOI:** 10.1186/s12883-022-02608-2

**Published:** 2022-03-05

**Authors:** Shoichi Tokumoto, Masahiro Nishiyama, Hiroshi Yamaguchi, Kazumi Tomioka, Yusuke Ishida, Daisaku Toyoshima, Hiroshi Kurosawa, Kandai Nozu, Azusa Maruyama, Ryojiro Tanaka, Kazumoto Iijima, Hiroaki Nagase

**Affiliations:** 1 Department of Pediatrics, Kobe University Graduate School of Medicine, 7-5-2 Kusunoki-cho, Chuo-ku, Kobe, Hyogo 650-0017 Japan; 2 Department of Neurology, Hyogo Prefectural Kobe Children’s Hospital, 1-6-7 Minatojima-minamimachi, Chuo-ku, Kobe, Hyogo 650-0047 Japan; 3 Department of Emergency and General Pediatrics, Hyogo Prefectural Kobe Children’s Hospital, 1-6-7 Minatojima-minamimachi, Chuo-ku, Kobe, Hyogo 650-0047 Japan; 4 Division of Pediatric Critical Care Medicine, Hyogo Prefectural Kobe Children’s Hospital, 1-6-7 Minatojima-minamimachi, Chuo-ku, Kobe, Hyogo 650-0047 Japan

**Keywords:** Febrile seizure, Clinical protocol, Anticonvulsant, Barbiturate, Benzodiazepine, Phenytoin

## Abstract

**Background:**

Febrile status epilepticus is the most common form of status epilepticus in children. No previous reports compare the effectiveness of treatment strategies using fosphenytoin (fPHT) or phenobarbital (PB) and those using anesthetics as second-line anti-seizure medication for benzodiazepine-resistant convulsive status epilepticus (CSE). We aimed to examine the outcomes of various treatment strategies for febrile convulsive status epilepticus (FCSE) in a real-world setting while comparing the effects of different treatment protocols and their presence or absence.

**Methods:**

This was a single-center historical cohort study that was divided into three periods. Patients who presented with febrile convulsive status epilepticus for ≥60 min even after the administration of at least one anticonvulsant were included. During period I (October 2002–December 2006), treatment was performed at the discretion of the attending physician, without a protocol. During period II (January 2007–February 2013), barbiturate coma therapy (BCT) was indicated for FCSE resistant to benzodiazepines. During period III (March 2013–April 2016), BCT was indicated for FCSE resistant to fPHT or PB.

**Results:**

The rate of electroencephalogram monitoring was lower in period I than period II+III (11.5% vs. 85.7%, *p*<0.01). Midazolam was administered by continuous infusion more often in period I than period II+III (84.6% vs. 25.0%, *p*<0.01), whereas fPHT was administered less often in period I than period II+III (0% vs. 27.4%, *p*<0.01). The rate of poor outcome, which was determined using the Pediatric Cerebral Performance Category scale, was higher in period I than period II+III (23.1% vs. 7.1%, *p*=0.03). The rate of poor outcome did not differ between periods II and III (4.2% vs. 11.1%, *p*=0.40).

**Conclusions:**

While the presence of a treatment protocol for FCSE in children may improve outcomes, a treatment protocol using fPHT or PB may not be associated with better outcomes.

## Background

Status epilepticus (SE) is one of the most common neurological emergencies in children, with an incidence of 18 to 41 cases per 100,000 children per year [1]. The mortality rate is 2.7–8%, and the rate of sequelae (mainly neurological) is 10–20% [2]. Prolonged febrile convulsions are the most common etiology of SE in childhood [3]. Febrile status epilepticus (FSE) is the most common form of SE in children, which accounts for 25% of all childhood SE [4]. Regarding treatment, various guidelines have been published targeting convulsive status epilepticus (CSE) and these guidelines recommend benzodiazepines as the first-line treatment for CSE [5–8]. In cases of resistance to benzodiazepines, non-anesthetic anticonvulsants are the primary choice. For example, the American Epilepsy Society recommends fosphenytoin (fPHT), valproic acid, or levetiracetam as second-line therapies, and, if these agents are not available, it recommends phenobarbital (PB) [5]. There is no clear evidence on additional treatments when cases are resistant to these second-line therapies, but usually anesthetic therapies are employed [5–7]. Although treatment protocols for CSE in children are currently being developed in many countries and communities, there have been no reports examining the effects of the presence or absence of such protocols on treatment outcomes. In addition, there are no reports comparing the effectiveness of treatment strategies using fPHT or PB and those using anesthetics as second-line anti-seizure medication for benzodiazepine-resistant CSE.

Therefore, we examined real-world comparative effects of different treatment strategies for febrile CSE (FCSE) on patient outcomes, focusing on two points as follows: (1) comparison of the effects of the presence or absence of a protocol, and (2) comparison of the effects of different protocols. Our main hypothesis was that the presence of a treatment protocol would improve outcomes in children with FCSE. In addition, we hypothesized that protocols using fPHT or PB would lead to better outcomes than those without fPHT or PB, and that protocols using fPHT or PB would reduce the number of times anesthetic therapy is administered to children with FCSE.

## Methods

### Study design

This was a single-center historical cohort study that was divided into three periods. We used data gathered in the framework of a prospectively collected medical database of consecutive cases admitted to Hyogo Prefectural Kobe Children’s Hospital, Japan, with convulsive seizures accompanied by fever, and reviewed related clinical charts.

### Definitions

SE was defined as a continuous seizure that lasted for at least 5 min, or a sequence of intermittent seizures without full recovery of consciousness between seizures [9]. CSE was defined as a seizure with overt motor symptoms longer than 30 min or recurrent seizures that lasted for a total of more than 30 min without the patient fully regaining consciousness [10]. FCSE was defined as a condition of febrile convulsive SE that persisted for 60 min or longer even after the administration of at least one anticonvulsant (i.e., benzodiazepine).

### Participants

We extracted the records of pediatric patients (aged <18 years) admitted to Hyogo Prefectural Kobe Children’s Hospital for convulsive seizures with fever and met the definition of FCSE between October 2002 and April 2016. We excluded the records of children who had a history of neurological problems before each hospitalization. The records of children with multiple hospitalizations were also excluded except for the first hospitalization episode. Children diagnosed with encephalitis, such as acute disseminated encephalomyelitis [11], limbic encephalitis [12], and pleocytosis in the cerebrospinal fluid (> 8 cells/μL), were excluded. Children with multiple organ failure were also excluded.

### Procedures

**Fig. 1:**
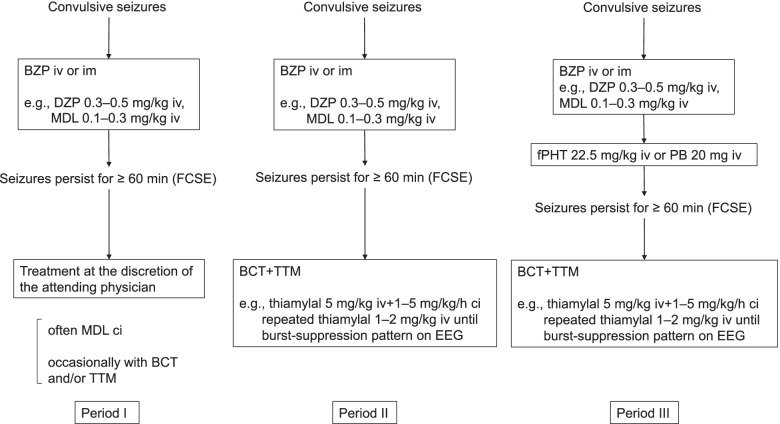
Treatment protocol for each period. BZP: benzodiazepine, DZP: diazepam, MDL: midazolam, FCSE: febrile convulsive status epilepticus, fPHT: fosphenytoin, PB: phenobarbital, BCT: barbiturate coma therapy, TTM: targeted temperature management, iv: intravenous, im: intramuscular, ci: continuous infusion, EEG: electroencephalogram.

The time between October 2002 and April 2016 was divided into three periods (Fig. 1). During period I (October 2002–December 2006), treatment was administered at the discretion of the attending physician, without a protocol. In practice, FCSE has often been treated with midazolam continuous infusion (MDL ci), without electroencephalogram (EEG) monitoring. There were no common rules among physicians regarding the timing of therapeutic interventions in period I. During period II (January 2007–February 2013), barbiturate coma therapy (BCT) was indicated for FCSE that was resistant to first-line drugs (mainly benzodiazepines). The protocol which had been used in period II was revised because PB and fPHT became available in Japan. After that, during period III (March 2013–April 2016), BCT was indicated for FCSE resistant to second-line drugs (fPHT or PB). During period II and III, the protocol was decided based on a consensus among pediatric neurologists, acute care physicians, and intensivists. Thiamylal was mainly used for BCT. During period I, targeted temperature management (TTM) was sometimes implemented at the discretion of the attending physician. During periods II and III, FCSE was treated with TTM when BCT was indicated. TTM was performed in accordance with previous reports [13]. Briefly, TTM controls body temperature using a cooling blanket (target 34°C or 36°C) with ventilation, muscle relaxant drugs, and vasopressors, as needed. Continuous EEG monitoring was also performed during BCT.

### Outcomes

The primary outcome was determined using the Pediatric Cerebral Performance Category (PCPC) scale score at the time of the last medical examination, within 90 days after onset. PCPC scale score was determined by pediatric neurologists. A PCPC scale score ≥2 was defined as a poor outcome. The PCPC scale is scored as follows: 1, normal; 2, mild disability; 3, moderate disability; 4, severe disability; 5, coma or vegetative state; 6, brain death [14]. The secondary outcomes were final diagnosis and ventilator-associated pneumonia (VAP) as treatment complications. The final diagnosis of FCSE consisted of acute encephalopathy (AE) or febrile seizure. The diagnosis of AE, such as AE with biphasic seizures and late reduced diffusion (AESD), or clinically mild encephalitis/encephalopathy with a reversible splenial lesion, was based on published literature [15]. The diagnosis of VAP was made using guidelines of the Infectious Disease Society of America [16]. Periods I and II+III were compared to investigate the effects of the presence or absence of a treatment protocol. In addition, periods II and III were compared to investigate the effects of different protocols, with or without second-line drugs, on FCSE that was resistant to first-line drugs.

### Data analyses

Results are expressed as number (%) or median (interquartile range [1st quartile, 3rd quartile]). Categorical variables were assessed using the Chi-square and Fisher's exact tests. Continuous variables were assessed using unpaired t-tests. For non-normally distributed continuous variables, the Mann–Whitney U test was used. All statistical analyses were performed using EZR, version 1.41 (Saitama Medical Center, Jichi Medical University, Saitama, Japan). A *p*-value <0.05 was considered statistically significant.

## Results

**Fig. 2:**
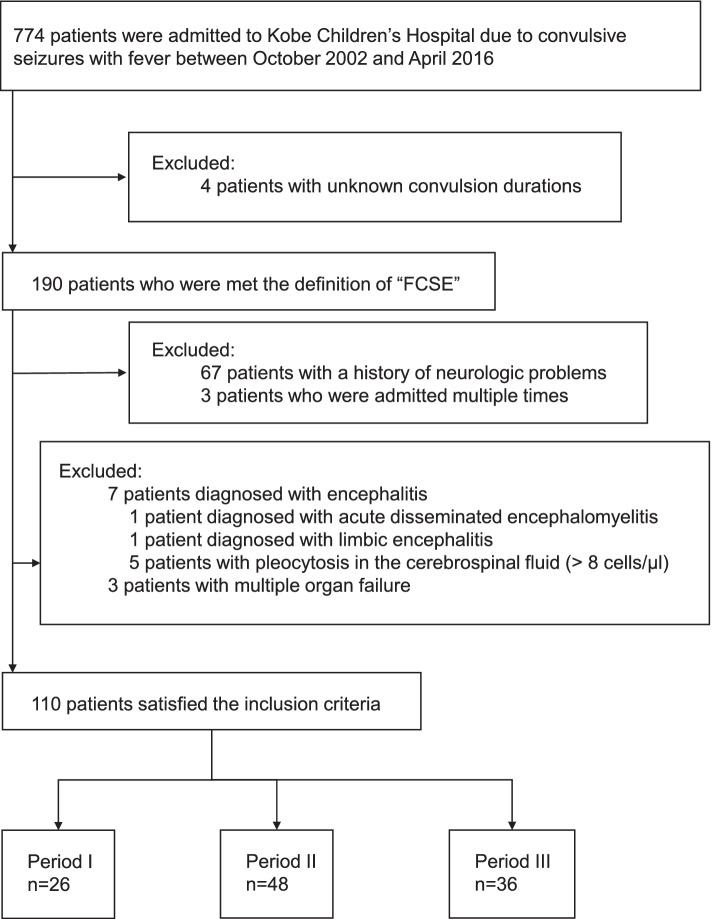
Selection of patients. The flowchart details the patient selection procedure. FCSE: febrile convulsive status epilepticus

From October 2002 to April 2016, 774 patients were hospitalized due to convulsive seizures with fever (Fig. 2). Of these, 190 patients met the definition of FCSE. From this group, 67 patients with a history of neurological disorders, three patients with multiple hospitalizations, seven patients with encephalitis, and three patients with multiple organ failure were excluded. Ultimately, 110 patients were included in the study. Periods I, II, and III comprised 26, 48, and 36 patients, respectively.

### Comparison of the effects of the presence or absence of a protocol (period I vs. period II+III)

Demographic and clinical characteristics of patients are shown in Table 1. The median patient age was significantly lower in period I (16.5 [10, 29.5] months) than in period II+III (22 [16, 39.25] months) (*p*=0.03). The median convulsive seizure duration was 150 (96.25, 345) min during period I and 133.5 (88.5, 211.25) min during period II+III (*p*=0.30). The number of patients who underwent EEG was significantly higher in period II+III than in period I (*p*<0.001). The goals of treatment with EEG monitoring were as follows: in period I, suppression of ictal-interictal continuum on EEG for two patients and absence of clinical seizures for the other 24 patients; in period II+III, complete burst suppression on EEG for 29 patients, suppression of ictal-interictal continuum on EEG for 43 patients, and absence of clinical seizures for the remaining 12 patients. The number of patients who underwent intubation was 15 (57.7%) in period I and 41 (48.8%) in period II+III (*p*=0.57). Eleven (42.3%) patients underwent TTM during period I and 38 (45.2%) during period II+III (*p*=0.97). Among the antiepileptic drugs used, MDL ci was used more often in period I, while fPHT was used more often in period II+III (*p*<0.001).

**Table 1:** Demographic and clinical characteristics of patients by period

	Period I *n*=26	Period II+III *n*=84	*p*-value	Period II *n*=48	Period III *n*=36	*p*-value
Sex, male, n (%)	13 (50.0)	38 (45.2)	0.84	23 (47.9)	15 (41.7)	0.73
Age, months	16.5 (10, 29.5)	22 (16, 39.25)	0.03^b^	24 (15, 47.5)	20.5 (16, 35)	0.45^b^
History of febrile seizure, n (%)	3 (11.5)	21 (25.0)	0.18^a^	11 (22.9)	10 (27.8)	0.80
Temperature on admission, °C	39.1 (38.4, 39.8)	38.65 (38.1, 39.4)	0.20	38.65 (38.1, 39.4)	38.65 (38.1, 39.4)	0.69
Convulsive seizure duration	150 (96.3, 345)	133.5 (88.5, 211.3)	0.30^b^	125 (89.75, 210)	142.5 (84, 225.3)	0.81^b^
Duration of hospital stay, days	9.5 (4.75, 19.8)	7.5 (5, 13)	0.17^b^	7 (4, 12.3)	9 (5, 13)	0.21^b^
Period from onset to determination of outcome, days	26 (20, 39.8)	32 (27, 38.3)	0.84	31 (17.8, 37.8)	35.5 (30, 38.3)	0.28
EEG monitoring, n (%)	3 (11.5)	72 (85.7)	<0.001^a^	39 (81.3%)	33 (91.7%)	0.22^a^
Intubation, n (%)	15 (57.7)	41 (48.8)	0.57	23 (47.9)	18 (50.0)	1
TTM, n (%)	11 (42.3)	38 (45.2)	0.97	20 (41.7)	18 (50.0)	0.59
Vasopressor, n (%)	13 (50.0)	37 (44.0)	0.76	20 (41.7)	17 (47.2)	0.78
Number of antiepileptic drugs used	3 (2, 3)	2 (2, 3)	0.43^b^	2 (2, 3)	3 (2, 4)	0.07^b^
Details of antiepileptic drugs used						
DZP, n (%)	25 (96.2)	74 (88.1)	0.45^a^	44 (91.7)	30 (83.3)	0.31^a^
MDL, n (%)	19 (73.1)	69 (82.1)	0.47	38 (79.2)	31 (86.1)	0.59
fPHT iv, n (%)	0 (0)	23 (27.4)	0.002^a^	3 (6.3)	20 (55.6)	<0.001^a^
PB, n (%)	9 (34.6)	15 (17.9)	0.12	10 (20.8)	5 (13.9)	0.59
MDL ci, n (%)	22 (84.6)	21 (25.0)	<0.001^a^	19 (39.6)	2 (5.6)	<0.001^a^
Thiamylal ci, n (%)	9 (34.6)	38 (45.2)	0.47	20 (41.7)	18 (50.0)	0.59
MDL+Thiamylal ci, n (%)	9 (34.6)	10 (11.9)	0.02	10 (20.8)	0 (0)	0.004^a^
Time from onset to BCT initiation, h	5.5 (3.9, 37.1)	6.8 (5.0, 9.0)	0.97^b^	6.3 (5.3, 9.0))	6.9 (4.9, 8.9)	0.87^b^

Data are shown as the median (interquartile range) unless otherwise noted

*EEG* electroencephalogram, *TTM* targeted temperature management, *DZP* diazepam, *MDL* midazolam, *fPHT* fosphenytoin, *PB* phenobarbital, *BCT* barbiturate coma therapy, *iv* intravenous, *ci* continuous infusion

^a^: Fisher's exact test

^b^: Mann–Whitney U test

others: Chi-square test for categorical variables and t-test for continuous variables

The outcomes of these effects are shown in Table 2. The rate of poor outcomes (PCPC scale score ≥2) was significantly higher in period I than in period II+III. In period I, all three patients with EEG monitoring had good outcomes (PCPC scale score = 1). Of 23 patients without EEG monitoring, 17 had good outcomes (PCPC scale score = 1), and six had poor outcomes (PCPC scale score: 2 for three patients, 3 for two patients, and 4 for one patient). In period II+III, of 72 patients with EEG monitoring, 64 had good outcomes (PCPC scale score = 1), and eight had poor outcomes (PCPC scale score: 2 for four patients, 3 for three patients, and 4 for one patient). All 12 patients without EEG monitoring had good outcomes (PCPC scale score = 1). The rate of AE was significantly higher in period I than in period II+III. The rate of VAP was 0% (0 out of 26) in period I, and 17.1% (7 out of 84) in period II + III (*p*=0.20).

**Table 2:** Outcome comparison, period I vs. period II+III

	Period I *n*=26	Period II+III *n*=84	Risk difference (95% CI)	*p*-value
PCPC scale score ≥2, n (%)	6 (23.1)	6 (7.1)	-16.0% (-30.0% to -1.8%)	0.03
Final diagnosis				
AE, n (%)	9 (34.6)	11 (13.1)	-21.5% (-39.5% to -3.9%)	0.02
AESD, n (%)	5 (19.2)	6 (7.1)	-12.1% (-26.0% to 0.8%)	0.12
MERS, n (%)	0 (0)	1 (1.2)	1.2% (-2.8% to 1.2%)	1
Unclassified AE, n (%)	4 (15.4)	4 (4.8)	-10.6% (-21.8% to 0.6%)	0.09
Febrile seizure, n (%)	17 (65.4)	73 (86.9)	21.5% (3.9% to 39.5%)	0.02
Treatment-related complication				
VAP, n (%)	0 (0)	7 (8.3)	8.3% (-3.5% to 8.3%)	0.20

*CI* confidence interval, *PCPC* Pediatric Cerebral Performance Category, *AE* acute encephalopathy, *AESD* acute encephalopathy with biphasic seizures and late reduced diffusion, *MERS* clinically mild encephalitis/ encephalopathy with a reversible splenial lesion, *VAP* ventilator-associated pneumonia

### Comparison of the effects of different protocols (period II vs. period III)

The median convulsive seizure duration was 125 (89.75, 210) min during period II and 142.5 (84, 225.25) min during period III (*p*=0.81). The number of patients who underwent EEG was similar in periods II and III (*p*=0.38). The goals of treatment with EEG monitoring were as follows: in period II, complete burst suppression on EEG for 11 patients, suppression of ictal-interictal continuum on EEG for 28 patients, and absence of clinical seizure for the remaining nine patients; in period III, complete burst suppression on EEG for 18 patients, suppression of ictal-interictal continuum on EEG for 15 patients, and absence of clinical seizure for the remaining three patients. The number of patients who underwent intubation was 23 (47.9%) in period II and 18 (50.0%) in period III (*p*=1). Twenty (41.7%) patients underwent TTM during period II and 18 (50.0%) during period III (*p*=0.59). Among the antiepileptic drugs, MDL ci was used more often in period II, and fPHT was used more often in period III.

The outcomes of this comparison are shown in Table 3. There were no differences between the two groups in the rate of poor outcomes (PCPC scale score ≥2) or the rates of final diagnoses. The rate of VAP was 6.3% (3 out of 48) in period II and 11.1% (4 out of 36) in period III (*p*=0.46).

**Table 3:** Outcome comparison, period II vs. period III

	Period II *n*=48	Period III *n*=36	Risk difference (95% CI)	*p*-value
PCPC scale score ≥2, n (%)	2 (4.2)	4 (11.1)	6.9% (-3.5% to 13.8%)	0.40
Final diagnosis				
AE, n (%)	7 (14.6)	4 (11.1)	-3.5% (-14.5% to 10.8%)	0.75
AESD, n (%)	3 (6.2)	3 (8.3)	2.1% (-6.9% to 11.1%)	1
MERS, n (%)	1 (2.1)	0 (0)	-2.1 (-2.1% to 1.8%)	1
Unclassified AE, n (%)	3 (6.3)	1 (2.8)	-3.5% (-7.4% to 5.1%)	0.63
Febrile seizure, n (%)	41 (85.4)	32 (88.9)	3.5% (-10.8% to 14.5%)	0.75
Treatment-related complication				
VAP, n (%)	3 (6.3)	4 (11.1)	4.9% (-5.8% to 13.9%)	0.46

*CI* confidence interval, *PCPC* Pediatric Cerebral Performance Category, *AE* acute encephalopathy, *AESD* acute encephalopathy with biphasic seizures and late reduced diffusion, *MERS* clinically mild encephalitis/ encephalopathy with a reversible splenial lesion, *VAP* ventilator-associated pneumonia

## Discussion

To the best of our knowledge, this is the first report comparing the neurological outcome before and after the implementation of treatment protocols for FCSE in children. This study suggests that treatment protocols for FCSE in children improve the outcomes. In contrast, treatment protocols using fPHT or PB were not associated with better outcomes nor avoidance of anesthetic therapy in children with FCSE.

The efficacy of second-line drugs such as fPHT, valproate, and levetiracetam has been reported to be similar in patients with benzodiazepine resistant SE [17]. Moreover, we have previously reported that there is no difference in the efficacy of fPHT and MDL ci in pediatric patients with benzodiazepine resistant SE [18]. Previous studies showed that the efficacy of several second-line agents was similar in patients with benzodiazepine resistant SE, including febrile SE. However, the effects with or without treatment protocolsor those due to differences in protocols in a real-world setting are yet to be clarified.

### Comparison of the effects of the presence or absence of a protocol (period I vs period II+III)

There are several reports on the usefulness of treatment protocols for SE in children [19, 20]. Xie et al. [19] reported that before and after the implementation of a protocol, the time between the first detection of seizures and the administration of first- and second-line drugs was significantly reduced. Cassel et al. [20] also reported that the time to second-line drug administration was significantly reduced by the implementation of a protocol. Furthermore, it has previously been reported that early treatment of SE might affect outcomes. Gainza-Lein et al. [21] reported that all deaths in pediatric SE were when benzodiazepines could not be administered within 10 min from seizure onset. Herein, the median time from seizure onset to BCT initiation was similar between periods I and II+III. Nevertheless, during period I, there were few patients in whom BCT was started more than 24 h after seizure onset (range, 2.5–85.2 h). In period II + III, BCT was started within 24 h after onset in all patients (range, 2.1–21.3 h). In period I, BCT was started more than 24 h after onset in three patients, and the outcomes of two of these patients were poor. As reported in previous studies, implementation of a protocol for SE enables early treatment, and this can improve outcomes. In the present study, these reasons may also potentially contribute to the improved outcomes.

Although there was no standard protocol in period I, in practice many patients were treated with MDL ci without EEG monitoring to achieve termination of clinical seizures. In a previous study, 164 patients with SE underwent continuous EEG monitoring, and 48% of them had persistent electrographic seizures, even after the apparent seizures were suppressed [22]. Nagase et al. [23] reported that five out of eight children who received MDL ci for refractory FSE had breakthrough seizures, and concluded that clinical seizure control using MDL ci without EEG monitoring is insufficient to prevent neurological damage. Our results might potentially be explained by the presence or absence of EEG monitoring. Compared to period I, the use of EEG monitoring increased in periods II and III. Moreover, the goals of treatment had been changed from clinical seizure control to complete burst suppression or suppression of ictal-interictal continuum on EEG. Therefore, we speculate that those reliable seizure control for SE may have contributed to the improved outcome.

The rate of final diagnosis of AE was significantly higher in period I than in period II+III. Tighter control of seizures with EEG monitoring might have suppressed excitotoxicity and the development of AE. Indeed, prolonged seizures cause excitotoxicity [24], and the association of excitotoxicity with the pathophysiology of AE, such as AESD, has been reported [25, 26].

In the present study, the median patient age was lower in period I than in period II+III. In a systematic review of outcomes from pediatric CSE, younger patients had higher rates of sequelae and mortality [10]. The poorer outcomes of patients in period I than those of patients in period II+III in our study may be due to the younger age of the patients.

### Comparison of the effects of different protocols (period II vs period III)

The outcomes were not different between treatment protocols with and without fPHT or PB. We expected better outcomes in period III than period II, and the use of second-line drugs to reduce the number of cases of BCT, resulting in a reduction of VAP, a complication of mechanical ventilation. However, the use of second-line drugs did not improve outcomes or reduce the VAP incidence.

In period II, the proportion of patients receiving MDL ci was higher than that in period III. However, contrary to treatment in period I, treatment in periods II and III was performed under EEG monitoring. This allowed breakthrough seizures to be detected, which might explain the lack of differences in outcome. Treatment using fPHT occurred more frequently in period III than in period II. As a second-line treatment for benzodiazepine-resistant SE, fPHT is used worldwide [5–8]. However, its effect on febrile seizures is still unknown, and some reports have shown negative effects. Olson et al. [27] investigated the preventive effects of PB, valproic acid, and phenytoin on hyperthermia-induced seizures in rat pups. Animals injected with PB and valproic acid had seizure temperature thresholds that were significantly higher than those of controls, while animals injected with phenytoin had seizure temperature thresholds that were equal to or lower than those of controls. Among 56 patients experiencing 62 episodes of childhood FSE, phenytoin administration terminated seizures in nine (14.5%) episodes and failed to terminate them in 25 (40.3%) episodes [28]. Atypical febrile seizures, including SE, are reported to be associated with sodium channel mutations [29–31]. Phenytoin, a sodium channel blocker, may have little effect on FSE [28]. In our study, during period III, fPHT was used mainly as a second-line drug. The lack of difference in outcomes between periods II and III may be due to the poor effect of fPHT on FSE. In recent years, studies have reported that the therapeutic effect of levetiracetam on SE is equivalent to that of phenytoin [32, 33]. Reductions in the severity and mortality of hyperthermic seizures have been observed in levetiracetam-treated rats with cortical dysplasia, where levetiracetam exerted protective effects against hyperthermic seizure-induced blood-brain barrier disruption [34]. In childhood FSE outcomes may be improved by using levetiracetam instead of phenytoin as a second-line drug.

Outcomes of SE are affected by pre-existing neurological abnormalities [35, 36], but a major strength of the present study is that it only targeted patients without pre-existing neurological abnormalities. However, this study also has some limitations. First, it was an observational study without controls, based at a single center. Second, although the data were extracted from a prospectively collected database, the study itself was retrospective. It is unlikely that all of the listed exclusion criteria were documented accurately in every patient. Third, this study targeted febrile seizures, not afebrile seizures such as epilepsy and hypoxic-ischemic encephalopathy. Therefore, the results are not applicable to seizures caused by other factors. Finally, we did not have data on the doses of each antiepileptic drug. As a previous systematic review of intensive care treatment of refractory SE in children, the use of EEG monitoring resulted in the need for high doses of midazolam for treatment aimed at controlling electrographic seizures [37]. Using EEG monitoring, the goal of treatment changed from absence of clinical seizures in period I to suppression of ictal-interictal continuum or complete burst suppression on EEG in periods II and III. The doses of antiepileptic drugs may have changed as the goal of treatment changed, but this study did not reveal them.

## Conclusions

Our study showed that the presence of a treatment protocol for FCSE in children may improve patient outcomes. Furthermore, our results suggest that a treatment protocol that includes the use of fPHT or PB may not be associated with better outcomes or help to elude the need for anesthetic therapy in these children.

## Data Availability

The data sets in this study are available from the corresponding author upon reasonable request.

## Abbreviations

SE: Status epilepticus
CSE: Convulsive status epilepticus
FSE: Febrile status epilepticus
FCSE: Febrile convulsive status epilepticus
fPHT: Fosphenytoin
PB: Phenobarbital
MDL ci: Midazolam continuous infusion
BCT: Barbiturate coma therapy
TTM: Targeted temperature management
EEG: Electroencephalogram
PCPC: Pediatric Cerebral Performance Category
VAP: Ventilator-associated pneumonia
AE: Acute encephalopathy
AESD: Acute encephalopathy with biphasic seizures and late reduced diffusion
